# Estrogenic Action in Stress-Induced Neuroendocrine Regulation of Energy Homeostasis

**DOI:** 10.3390/cells11050879

**Published:** 2022-03-03

**Authors:** Kristen N. Krolick, Haifei Shi

**Affiliations:** 1 Physiology and Neuroscience Program, Department of Biology, Miami University, Oxford, OH 45056, USA; kristen.krolick@cchmc.org; 2 Center for Autoimmune Genomics and Etiology, Cincinnati Children’s Hospital Medical Center, Cincinnati, OH 45229, USA

**Keywords:** estrogen, estrogen receptor, hypothalamic-pituitary-gonadal axis, hypothalamic-pituitary-adrenal axis, stress

## Abstract

Estrogens are among important contributing factors to many sex differences in neuroendocrine regulation of energy homeostasis induced by stress. Research in this field is warranted since chronic stress-related psychiatric and metabolic disturbances continue to be top health concerns, and sex differences are witnessed in these aspects. For example, chronic stress disrupts energy homeostasis, leading to negative consequences in the regulation of emotion and metabolism. Females are known to be more vulnerable to the psychological consequences of stress, such as depression and anxiety, whereas males are more vulnerable to the metabolic consequences of stress. Sex differences that exist in the susceptibility to various stress-induced disorders have led researchers to hypothesize that gonadal hormones are regulatory factors that should be considered in stress studies. Further, estrogens are heavily recognized for their protective effects on metabolic dysregulation, such as anti-obesogenic and glucose-sensing effects. Perturbations to energy homeostasis using laboratory rodents, such as physiological stress or over-/under- feeding dietary regimen prevalent in today’s society, offer hints to the underlying mechanisms of estrogenic actions. Metabolic effects of estrogens primarily work through estrogen receptor α (ERα), which is differentially expressed between the sexes in hypothalamic nuclei regulating energy metabolism and in extrahypothalamic limbic regions that are not typically associated with energy homeostasis. In this review, we discuss estrogenic actions implicated in stress-induced sex-distinct metabolic disorders.

## 1. Introduction

Characteristic sex differences in energy metabolism and its dysregulation, induced by psychological stressors (e.g., restraint stress) and dietary stressors, such as under-feeding (e.g., dieting and fasting) and over-feeding (e.g., feeding a high-fat diet [HFD]), are witnessed in humans [1,2,3]. Besides regulating reproductive characteristics and functions, estrogens account for many sex differences in energy balance via regulating feeding behavior and energy metabolism [4]. Estrogens are heavily recognized for their protective effects on metabolism witnessed in both women and men [4]. In this review, we explain effects of estrogens on neuroendocrine regulation of energy homeostasis. Because one of the best ways to understand mechanisms of energy homeostasis in a laboratory setting is to perturb it, we discuss examples of stress-induced effects throughout. We generalize that stress-induced disturbance and threat to energy homeostasis, no matter the original stimulus, converge on activation of the hypothalamic–pituitary–adrenal (HPA) axis. Thus, we include specific examples from dietary stressors to psychological stressors. In order to comprehensively explain effects of estrogens on neuroendocrine regulation of energy homeostasis, we delve into fundamentals that refresh and expand readers’ understanding of the female reproductive cycles in humans and rodents, hormonal control of sexual differentiation of the brain, and the neurocircuitry underlying energy homeostasis. Although we utilize many examples involving rodents, understand feeding behavior and physical activities in humans are perhaps more complex due to influence by many non-biological factors such as culture and socioeconomic variables, which are not discussed in this review.

### 1.1. Overview of Neuroendocrine Regulation of Energy Homeostasis

Homeostasis of energy metabolism is critical for survival. Accordingly, the neurobiology underlying these processes is constantly adapting to reflect the homeostatic needs of individual organisms, food availability and demands, and food wanting and value. The central nervous system (CNS) communicates with various peripheral organs and tissues via sympathetic efferents (i.e., the splanchnic nerves) and parasympathetic efferents (i.e., the vagus nerve) to control multiple aspects of energy expenditure, intake, and digestion and absorption [5]. Vagal afferents can either be stimulated directly by gastrointestinal tract tension changes from food or indirectly by chemical stimuli activating taste receptors, and subsequent release of gastrointestinal peptides [5]. The released peptides may induce appetite (e.g., ghrelin) or satiety (e.g., gastric leptin, cholecystokinin, glucagon-like peptide 1, and peptide YY) [5]. Circulating nutrients influence feeding through brainstem signaling to hypothalamic circuits [5]. Hypothalamic circuits are canonically known as the homeostatic circuits of energy metabolism [6]. Other notable peptides and their receptors involved in hypothalamic circuits of feeding and energy metabolism known to have anorexigenic or orexigenic functions are cannabinoid receptor type 1 [7] and cocaine and amphetamine regulated transcript [8].

Feeding is not only guided by homeostatic energy needs which determine the number of calories ingested, but is also regulated by rewarding value of food comprising neuropeptide Y (NPY) [9] and dopamine [10] pathways in the hypothalamic and extrahypothalamic nuclei, which are associated with the sensory inputs of food such as smell and taste and modulated by physiological states such as hunger and satiety [11]. Control of energy homeostasis involves other aspects regulated by hypothalamic circuitry besides feeding, including regulation of lipid metabolism [12], distribution of adipose tissue [12], glucose metabolism [13], and insulin sensitivity [13]. Stress has been reported to alter feeding behavior, energy expenditure, and glucose and lipid metabolism [14].

### 1.2. Overview of Estrogen Regulation of Energy Homeostasis

Estrogens possess many different biological functions, and they are one of major factors underlying key metabolic and behavioral differences between men and women. One commonly cited indicator of how estrogen regulates metabolism is the prevalence of obesity and insulin resistance increasing among women after menopause when levels of endogenous estrogens decline [15]. For example, in an analysis of large-scale polling on a national level that included over 2500 American women, incidence of obesity increased from 37.0% in women 20–39 years of age to 44.6% in women 40–59 years of age [16]. Additionally, menopause is associated with increased central body fat accumulation in previously normal weight women, posited to be due to their declined estrogen levels [17]. Akin to postmenopausal compared with premenopausal women, ovariectomized rodents with depleted levels of circulating estrogen significantly increase their food intake and body weight gain [1,2,3]. Therefore, both human and animal studies support important roles of estrogen in metabolic regulation. Estrogens interact with estrogen receptors (ER) within brain regions underlying homeostatic regulation of energy metabolism have been studied to elucidate molecular mechanisms responsible for sex differences in energy homeostasis.

## 2. Energy Homeostasis across Reproductive Cycles Regulated by Estrogen

### 2.1. Reproductive Cycles in Humans and Rodents

**Figure 1:**
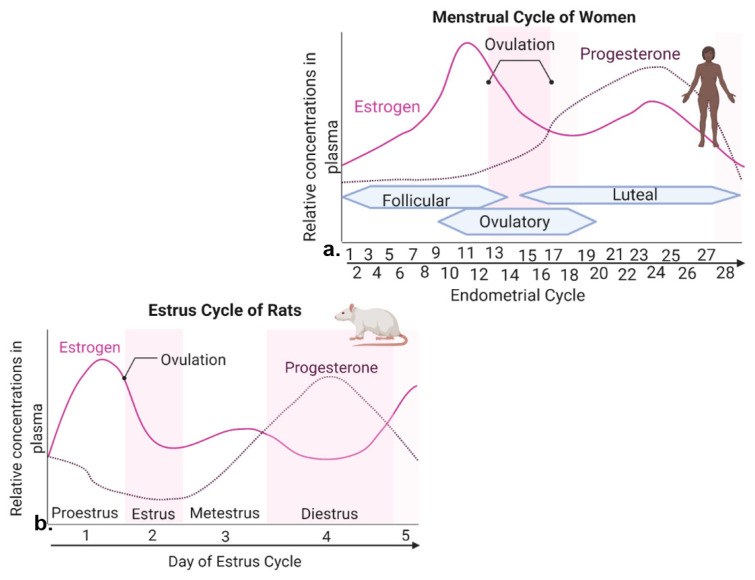
Levels of estrogen and progesterone during woman menstrual cycle and rodent estrous cycle. (**a**) The menstrual cycle of women takes place in ~28 days and consists of three prominent phases. The follicular phase is defined as the beginning of menstruation when shedding of the endometrium and bleeding occurs. During the beginning of this phase, estrogen and progesterone levels are low. Bleeding marks the start of a slight increase in follicular stimulating hormone (FSH) levels. FSH levels decrease and one follicle proceeds to develop, producing estrogen. The ovulatory phase is marked by a surge in luteinizing hormone (LH) and FSH, with ovulation occurring ~16–32 h post surge. Estrogen levels start to decrease, and progesterone increases. During the luteal phase, FSH and LH decrease, and the corpus luteum forms, which produces progesterone. Estrogen levels remain high, reaching its second largest peak. If fertilization does not occur, the corpus luteum degenerates, no longer producing progesterone, and estrogen levels decline, eventually causing the breakdown of uterus endometrium lining and the start of a new follicular phase. (**b**) The estrous cycle of rodents lasts ~4–5 days. Similar peaks of estrogen and progesterone are seen in menstrual cycle of women and estrous cycle of rats. This template was created by Nina Kessler of BioRender.com, and was edited using BioRender.com, accessed on 20 December 2021.

Rodent models are widely used in order to uncover molecular estrogen interactions in energy homeostasis. Therefore, it is worthwhile to briefly review woman and rodent reproductive cycles, noting their similarities and differences (Figure 1).

In women, the reproductive cycle is the menstrual cycle, also referred to as the endometrial cycle, which occurs ~28 days and consists of three prominent phases known as follicular, ovulatory, and luteal phases [18] (Figure 1a). The follicular phase distinguishes the beginning of menstruation, when shedding of the endometrium and bleeding occur [18]. During the beginning of this phase, the levels of estrogen and progesterone are low. Bleeding marks the start of a slight increase in follicular stimulating hormone (FSH) levels, which causes the development of follicles in the ovaries [18]. FSH levels then decrease and only one follicle proceeds to develop, which produces estrogen [18]. The ovulatory phase is marked by a surge in luteinizing hormone (LH) and FSH, causing ovulation (i.e., egg release) ~16–32 h post surge [18]. Estrogen levels start to decrease, and progesterone increases. During the luteal phase, levels of FSH and LH decrease, and the follicle forms into a corpus luteum, which produces progesterone and estrogen [18]. Estrogen levels are increased, reaching the second largest peak. High levels of estrogen and progesterone cause the uterus lining to thicken to prepare for possible implantation of a fertilized egg [18]. If fertilization does not occur, the corpus luteum degenerates, no longer producing progesterone, and as hormone levels decline, they eventually cause the breakdown of uterus endometrium lining and the start of a new follicular phase [18].

The rodent form of the reproductive cycle is called the estrous cycle [19], which occurs over a period of ~4–5 days. It consists of four main phases: proestrus, estrus, metestrus and diestrus [19] (Figure 1b), which correspond to three phases of the human menstrual cycle [19,20]. Notice that during both human and rodent cycles, as in many mammals, there are two large peaks in estrogen and one peak in progesterone levels (Figure 1) [18,19]. Specifically, in both rodents and women, the highest peak of estrogen proceeds ovulation [18,19]. The estrous cycle of rodents begins with proestrus, which is akin to the follicular phase in women that marks menstruation [19,20]. During proestrus, there is a rise in circulating estrogen and a small surge in prolactin, along with a rise in FSH and LH levels [19]. Proestrus is followed by a sharp decrease in estrogen levels and the start of ovulation, marking the estrous phase of rodents [19]. After estrus, metestrus and diestrus occur next, and are homologous to the early and late secretary phases of the human endometrial cycle, in which the progesterone peak takes place [19,20] (Figure 1). In summary, naturally cycling female rodents are avidly used laboratory models of women, with regular reproductive cycles across which similar feeding behavior and metabolic changes are witnessed in response to hormone fluctuation.

### 2.2. Energy Homeostasis across Female Reproductive Cycles

The reproductive cycles and related fluctuating levels of estrogens affect caloric intake and macronutrient selection differently. In many species including humans, caloric intake changes across reproductive cycles due to changes in meal size, with females eating the most calories when estrogen levels are low during metestrus, and eating the fewest calories when estrogen levels are high immediately prior to ovulation [17].

Interestingly, suppression of caloric intake by estrogen ceases when palatable food choices are offered alongside regular diet in female rhesus monkeys [21]. Food choice and macronutrient intake are also affected by the menstrual cycle in women. Self-assessed food craving and macro- and micro-nutrient intake in 259 healthy women was analyzed for a period of two complete menstrual cycles [22]. During the late luteal phase when estrogen levels are at their lowest level prior to menstruation, appetite and food craving for chocolate and sweet and salty flavors were higher, comparing with follicular and ovulatory phases when estrogen levels increase and peak during late follicular phase and beginning of ovulation [22]. Interestingly, total protein intake and percentage of caloric intake from protein were greater during the mid-luteal phase with the second peak of estrogen levels, comparing with the ovulatory phase following the first and higher peak of estrogen (Figure 1a) [22]. As discussed in more detail in Section 3 below, the reason for this owes to slower estrogen genomic actions via nuclear ERs [23]. The most prominent estrogen effects caused by estrogenic actions through its nuclear receptors occur during phases of relatively low levels of estrogen, if the proceeding phase has the peak estrogen level.

In summary, the relationship between circulating hormone levels, feeding behavior, and energy metabolism is complex, and estrogen effects are dynamic across the female cycles in women and experimental animals.

### 2.3. Female Cycles Regulated by Estrogen

**Figure 2:**
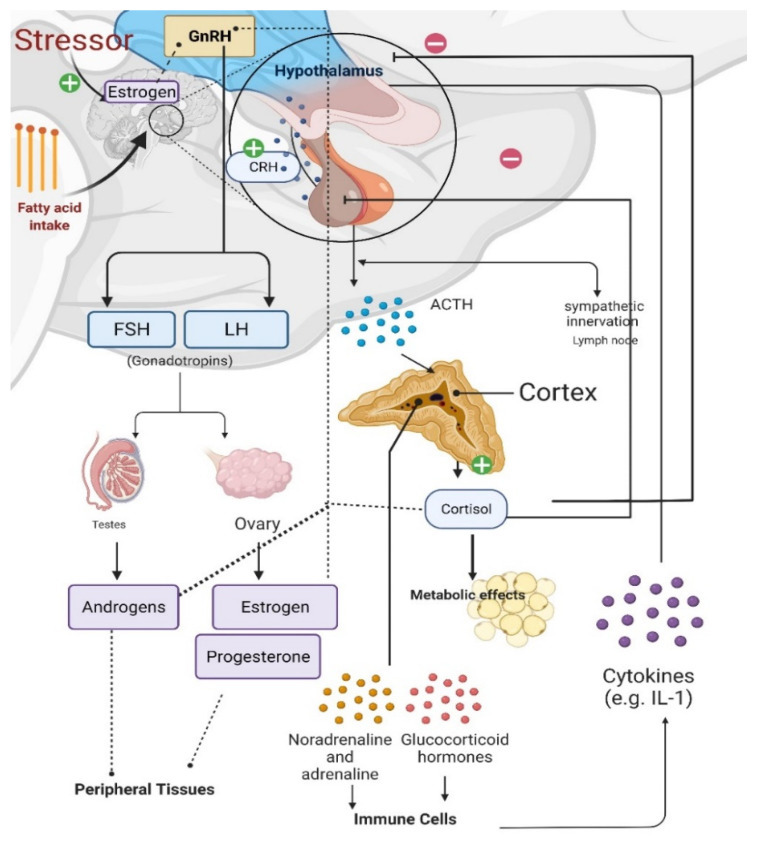
Effects of stress on the gonadal axis, immune cells, and metabolism. Gonadotropin releasing hormone (GnRH) release from the hypothalamus causes the anterior pituitary to secrete gonadotropins, luteinizing hormone [LH] and follicle-stimulating hormone [FSH]), which stimulate gonadal steroidogenesis. Estradiol synthesis and release from the ovaries regulates GnRH release. Estrogens can also be synthesized in the brain of males and females from androgens via aromatase in neurons; and estrogens can be synthesized in and have various effects at peripheral tissues. When the hypothalamic–pituitary–adrenal (HPA) axis is stimulated, corticotropin releasing hormone (CRH) from the hypothalamus triggers adrenocorticotropic hormone (ACTH) release from the anterior pituitary and increased cortisol production in the adrenal glands. Cortisol provides negative feedback to both the hypothalamus and pituitary; however, prolonged stress impairs this negative feedback regulation, causing long-lasting psychological and metabolic maladies. Prolonged fatty acid intake causes proliferative cytokines in the hypothalamus of males, which is protected in females. Original template edited with and reproduced from BioRender.com, accessed on 20 December 2021.

Estrogens plays critical roles in regulating great variety of physiological and behavioral functions. In females, the majority of estrogens, predominantly of the type 17β-estradiol (17β-E2) are synthesized in the periphery (i.e., ovaries) from cholesterol via the steroidogenic pathway involving a series of biochemical reactions [24]. Estrogen steroidogenesis also occurs in Leydig and germ cells of the testis in males, although levels of produced estrogen are relatively low [25]. In both males and females, gonadotropin releasing hormone (GnRH) from the hypothalamus triggers the anterior pituitary to secrete gonadotropins LH and FSH, which act on the gonads to stimulate steroidogenesis and gametogenesis [26] (Figure 2). In females, ovarian 17β-E2 synthesis and release from the ovaries negatively and positively regulates GnRH release in a pulsatile manner, although many effects of CNS-derived neuroestradiol on GnRH have been found to be more responsible for regulating GnRH (Figure 2) [26,27,28,29], discussed in more detail in Section 4 below.

Prolonged stress and increased glucocorticoid levels induce immune responses, elevate circulating cytokine levels, and dysregulate GnRH expression [26]. Therefore, the HPA axis, the hypothalamic-pituitary-gonadal (HPG) axis, and immune cells all interact with each other (Figure 2) [30,31]. During the HPA axis response to stress, corticotropin releasing hormone (CRH) is released from the hypothalamus, causing the release of adrenocorticotropic hormone (ACTH) from the anterior pituitary and subsequent production of cortisol (in rodents)/corticosterone (in humans) from the adrenal cortex [30,32]. Sympathetic activation during stress also regulates energy metabolism along with cardiovascular and pulmonary responses via sympathoneural (i.e., sympathetic innervation) and sympathoadrenomedullary (i.e., noradrenaline and adrenaline) systems (Figure 2) [33].

Major sex differences exist in stress-induced and stress-related metabolic disorders or disease states, pointing to differential interactions between the HPG axis, HPA axis, and immune cells [30,34,35]. For example, prolonged HFD feeding causes inflammation with elevated proinflammatory cytokines in the hypothalamus, hippocampus, and cortex of males but not females [36]. A recent experiment underlying female metabolic protection against HFD feeding has demonstrated peroxisome proliferator-activated receptor gamma coactivator 1 alpha (PGC-1α) as a regulator of ERα transcription [36]. Specifically, chronic HFD exposure for 16 weeks causes increased proinflammatory cytokines, but reduced PGC-1α and ERα transcription, in the arcuate nucleus of the hypothalamus (ARC) of male mice, causing a metabolic dysfunction phenotype of myocardial complications. Conversely, both PGC-1α knockout (KO) and ERα overexpression resulted in protection from these metabolic complications [36]. PGC-1α effects are likely to be upstream of ERα. Even in male mice, ERα can be protective against HFD feeding-related metabolic disorders. Novel regulators of ERα are still being discovered. Future studies that delve into the molecular details and underlying mechanisms of such sex differences await discovery.

## 3. Action of Estrogens via Estrogen Receptors

### 3.1. Isoforms of Natural Estrogens

Estrogens elicit a range of responses due to complex interactions between genomic and non-genomic estrogenic signaling [37]. In mammals, including humans, natural estrogens include a few steroid molecules with related biochemical structures, including estrone (E1), estradiol (E2; including 17β-E2 and its endogenous enantiomer 17α-E2), estriol (E3), and estetrol (E4). These estrogen isoforms are important regulators of female and male reproductive systems [38,39,40].

The amount of estrogens changes with age and body mass index. For example, circulating levels of estrogens are more than doubled in obese versus lean postmenopausal women [41]. In addition, the ratio of estrogen isoforms changes. During reproductive period before menopause, the most abundant estrogen isoform is 17β-E2 produced in the ovaries. Meanwhile, E1 is converted from adrenal androstenedione by aromatase mostly in adipose tissues, as well as in the brain and bone tissues [42]. Aromatization of androstenedione and E1 synthesis in enlarged adipose tissues almost double in obesity and E1 becomes the predominant estrogen isoform in the obese population [42]. After menopause, 17β-E2 synthesis is markedly reduced, whereas E1 synthesis increases and becomes the primary isoform of estrogens [43].

Estrogenic intracellular signaling and responses are mediated by one or more isoforms of estrogens binding to different subtypes of either nuclear or membrane ERs [37]. Lipophilic estrogens are steroids that could initiate two kinds of cellular responses. First, estrogens cross cell membrane, bind to their classical nuclear receptors [44], and induce changes in gene transcription and protein translation [45], which are relatively slow genomic responses taking hours to days [23]. Second, estrogens accumulate within cell membrane [46], bind to membrane associated ERs, initiate intracellular signaling events via production of second messengers and activation of cytosolic proteins and pathways, which are relatively rapid non-genomic responses occurring on a time frame of seconds to minutes [47]. Genomic mechanisms are relatively well characterized, whereas non-genomic estrogenic signaling is less well understood [47].

### 3.2. Estrogen Genomic Actions via Nuclear Estrogen Receptors

Genes for nuclear ERα and ERβ are located on distinct chromosomes [48,49,50,51]. Nuclear ERα was first characterized, its gene cloned [51], DNA sequenced [49], and crystal structure of its ligand-binding domain determined [52], using uterus and vaginal extracts from rats [53] before nuclear ERβ was sequenced [48]. Binding of estrogens to nuclear ERα and ERβ in cytosol and nuclei of target cells form estrogen-ER complexes, induce receptor conformational changes, ER inhibitory protein dissociation, and receptor dimerization, Subsequently, estrogen-ER complexes translocate to the nucleus, bind to estrogen responsive elements on promoter regions of estrogen- regulated genes, recruit co-activators or co-repressors, function as transcription factors, and finally lead to target gene transcription or change rate of gene expression [40,44,54]. Ultimately estrogen genomic actions control cellular responses, growth, differentiation, and many other functions [55]. Additionally, nuclear ERs could elicit transcriptional responses and regulate gene expression independent of estrogens but via interacting with other transcription factors [56]. Thus, estrogens mediate long-lasting genomic effects in estrogen-targeted cells via nuclear ERs. Bioactive 17β-E2, the predominant estrogen isoform during reproductive years, potently binds to nuclear ERα is [57], with a relative binding affinity (RBA) of 100% compared with 17α-E2 with an RBA of 3.68% [58]. Conversely, in one study, ERα binding affinities of 17α-E2 and 17β-E2 for ERα in brain tissue have been found to be similar [59]. The biosynthetic pathway of 17α-E2 synthesis is complex and not fully understood [59]. Other natural forms of estrogens bind nuclear ERα with much lower affinity [60], such as E1 (7.3% RBA [58]) and E3 (9.7% RBA [58]), and phytoestrogens found in certain environmental and food compounds (less than 1/1000 RBA than 17β-E2 [61]). Interestingly, 17β-E2 and certain phytoestrogens increase the binding affinity of ERα and ERβ to ERE, with 17β-E2 increasing the RBA to ERE by 50% for both ERα and ERβ, and phytoestrogens exhibiting a lower extent [60].

Genetic mouse models targeting the expression of ERα or ERβ have been developed to characterize their differential physiological functions [62]. Interestingly, obesity and diabetes-like metabolic dysfunctions are reported in both male and female ERα KO mice [63], but anti-diabetic phenotypes with improved glucose regulation are reported in ERβ KO mice [64]. These in vivo mice studies support different, almost opposite, physiological effects and metabolic functions of ERα and ERβ [65].

### 3.3. Estrogen Non-Genomic Actions via Membrane Estrogen Receptors

Some estrogenic effects are elicited rapidly within milliseconds to seconds [47], suggesting non-genomic actions of estrogens via membrane receptors besides their relatively slower genomic actions via nuclear receptors involving gene transcription and protein translation that typically take hours to complete [23]. Several membrane ERs (mERs) have been identified, including G protein-coupled ER (GPER) (such as GPR30 [66,67] and Gq-mER [68,69,70,71]), membrane subpopulations of ERs (such as mERα/β) [72,73]), and ER-X [74,75]. Interestingly, the RBA is different between nuclear and membrane ERs. For example, 17α-E2 is the primary isoform of endogenous estrogens of ER-X, especially in the brain [75].

The non-genomic estrogenic action via meERs has received a surge of interest and is one of the fastest emerging areas in the field of estrogen research. The rapid non-genomic effects of estrogens via mERs are elicited by multiple intracellular signaling pathways producing and activating various types of second messengers and protein kinases [66,70,76]. Particularly, following binding of mERs, second messengers such as intracellular Ca^2+^ and cyclic adenosine monophosphate (cAMP) are produced and associated protein kinase C and protein kinase A are activated [77,78,79], or phosphoinositide-3 kinase (PI3K)/Akt and RAS/mitogen-activated protein kinase (MAPK) are activated [55], all of which are key signaling pathways that play critical roles in the control of energy homeostasis. Besides natural endogenous estrogens, mERs are activated by pharmacological compounds with high affinity and selectivity, such as GPER agonists G1 [80,81] and antagonists G15 [82]. It is noteworthy that some reagents, such as tamoxifen and raloxifene, have high affinity to bind to, and thus activate, both nuclear and membrane ERs [72,83,84,85]. Genetic mouse models lacking GPERs have been developed to test their complicated physiological roles in vivo [86,87,88,89]. These pharmacological and genetic tools advance our understanding in non-genomic estrogenic effects and contributions of mER-mediated signaling, and may provide novel therapeutic targets for treating postmenopausal diseases, including cardiovascular protection, breast cancer metastasis, neural regulation of homeostatic functions and eating disorders, and osteogenesis in women [71,90].

## 4. Action of Estrogens in Periphery and CNS

### 4.1. Estrogenic Effects on Peripheral Tissues

In females, the majority of peripheral estrogens are synthesized in the ovaries and mostly play important endocrine roles in regulating secondary sexual characteristics and reproductive functions [91]. Additionally, peripheral estrogens are locally synthesized at estrogen-targeted tissues in females, including breast and uterus, to maintain reproductive functions [92,93]. Peripheral estrogens are also synthesized at multiple tissues and organs such as the liver, adrenal glands, and adipose tissue [24] and exert local paracrine effects unrelated to reproduction throughout the body [94]. For example, in the liver 17β-E2 has protective effects on metabolic regulation of glucose and lipid [95,96]. In the adrenal glands, estrogens reduce aldosterone synthesis and are responsible for the lowered blood pressure recordings in pre-menopausal women compared with age-matched men [97]. In adipose tissue, estrogens play a key role in inhibiting hypertrophic adipose tissue expansion [98], especially in postmenopausal women whose estrogens are mainly synthesized in adipose tissue following ovarian estrogen depletion [99]. Furthermore, estrogens play regulatory action in a variety of tissues that are not traditionally known as targets of estrogens, including tissues in the nervous, cardiovascular, skeletal and immune systems [100,101,102,103,104].

### 4.2. Estrogenic Effects on Central Tissues

Besides the periphery, estrogens are also synthesized within the brain in males and females [105]. The aromatase-expressing neuronal cells aromatize androgens into neuroestradiol, which exerts autocrine and paracrine effects [105], and functions as a neurotransmitter or neuromodulator to influence various brain regions to modulate brain development and behavior [106].

It has been extensively studied that circulating peripheral estrogens target to the hypothalamus to regulate sexual behavior, release of gonadotropins and prolactin from the pituitary, and regulate the stress responses [107]. Recently, the classical feedback loop, known for the last several decades, between GnRH release from the hypothalamus and ovarian hormone levels has been overturned by new discoveries of neuroestradiol roles [108]. In 2013, brain hypothalamic neurons were found to secrete 17β-E2 to influence release of GnRH [109]; and in 2017 it was discovered that hypothalamic neuroestradiol production, but not peripheral estrogens, is necessary for maintaining the LH surge responsible for inducing ovulation [108]. Furthermore, it was discovered recently that neuroestradiol in the ventral medial nucleus of the hypothalamus (VMH) is required in each sex for optimal metabolic and sex-specific signaling [110].

#### 4.2.1. Hypothalamic and Extrahypothalamic Brain Regions

**Figure 3:**
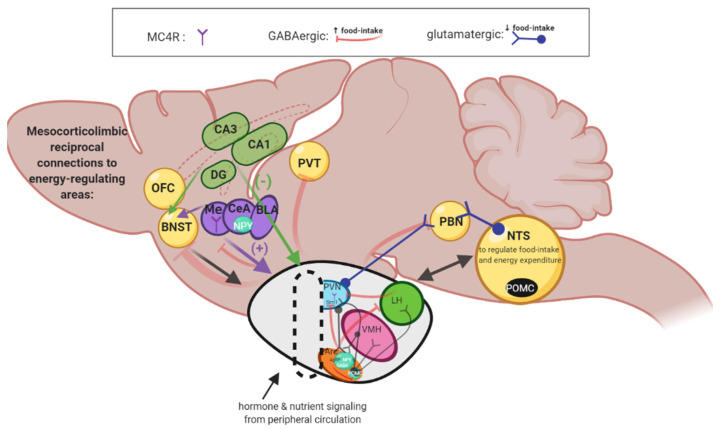
Neurocircuitry integrates hypothalamic brain regions in order to regulate food-intake behaviors, metabolic functioning, and stress responses, depicted in a rodent brain. The hypothalamus (gray), including the arcuate nucleus of the hypothalamus (ARC, orange), ventral medial nucleus of the hypothalamus (VMH, pink), lateral hypothalamus (LH, green), and paraventricular nucleus of the hypothalamus (PVN, blue). All hypothalamic subregions occur bilaterally (note: for clarity and simplicity, only unilateral nuclei are displayed) to the third ventricle (dashed oval) and have complex and reciprocal connections to brainstem and cortico-limbic brain regions. The ARC contains two major neuronal populations: the agouti-related protein (AgRP, top circle) orexigenic neurons, and the pro-opiomelanocortin (POMC, bottom circle with black) anorexigenic neurons. The PVN contains single-minded 1 (Sim1), and melanocortin-4 receptor (MCR4) neuronal populations which release neuropeptide Y (NPY), gamma-aminobutyric acid (GABA), and glutamate. Hippocampal regions include the hippocampal cornu ammonis 1 (CA1), hippocampal cornu ammonis 3 (CA3), and dentate gyrus (DG). Amygdala regions include the central nucleus of the amygdala (CEA), basolateral amygdala (BLA), and medial amygdala (Me). Other regions: bed nucleus of the stria terminalis (BNST), orbitofrontal cortex (OFC), parabrachial nucleus (PBN), nucleus tractus solitarius (NTS), paraventricular thalamic nucleus (PVT). Created with Biorender.com.

An adequate neurocircuit level understanding of integrated hypothalamic and corticolimbic region responses to energy homeostasis and feeding behavior (Figure 3) is necessary for understanding potential disruption of these systems by stress and for understanding the significance behind acknowledged sex differences. Briefly, ARC contains the orexigenic agouti-related protein (AgRP)/NPY pathway and the anorexigenic pro-opiomelanocortin (POMC)/α-melanocyte-stimulating hormone (α-MSH) pathway [6]. Specifically, activation of AgRP in the ARC releases gamma-aminobutyric acid (GABA) onto single-minded 1 (Sim1) neurons in the paraventricular nucleus of the hypothalamus (PVN) to increase food intake [111]. AgRP itself may also be released and is an endogenous antagonist of the melanocortin-4 receptors (MC4Rs) located on Sim1 neurons in the PVN [112]. Inhibitory GABAergic AgRP neurons of the ARC also promote feeding behavior, by projecting to neighboring POMC neurons and many other regions, such as the parabrachial nucleus (PBN), bed nucleus of the stria terminalis (BNST), lateral hypothalamic area, and medial amygdala [111,113,114,115] (Figure 3).

AgRP/NPY neurons synthesize and release orexigenic peptide NPY to increase food-intake [6]. Alternatively, anorexigenic POMC post-translational product, α-MSH, agonizes MC4Rs to decrease food intake [112] (Figure 3). When MC4Rs of Sim1 neurons in the PVN are agonized, Sim1 neurons activate glutamatergic projections to the PBN and decrease food intake [116]. Thus, noteworthy connections include the PVN projections to lateral PBN and the nucleus of the solitary tract (NTS) to inhibit food intake [117] (Figure 3). The PVN also plays a role in viscerosensory feedback to ingestive behavior. For example, the PVN can increase the response of the NTS to gastric distention causing satiation [118]. Indicative of the complex feedback loops within the hypothalamic circuit itself, the ARC POMC and AgRP neuronal populations project not only to the PVN, but to the anterior hypothalamic area (AHA) and VMH [119] to regulate feeding (Figure 3).

#### 4.2.2. Estrogenic Effects on Hypothalamic Nuclei Regulating Metabolism and Stress

Estrogens regulate energy homeostasis via ERα expressed in multiple hypothalamic nuclei (see review [120]). Here, we focus on the hypothalamic nuclei involved in stress responses.

The PVN houses both parvocellular and magnocellular neurons [121]. Parvocellular neurons exhibit both endocrine and autonomic functioning. For example, autonomic parvocellular neurons project to the dorsal motor nucleus of the vagus housing parasympathetic preganglionic cells and the intermediolateral cell column of the spinal cord housing sympathetic preganglionic cells [121]. These autonomic projections control important aspects of energy homeostasis such as pancreatic secretion, thermogenesis, lipid storage at adipose tissues, hepatic glucose flux, and peripheral glucose uptake [117,122,123]. Endocrine functions of parvocellular neurons in the medial PVN release peptides at the median eminence such as CRH, thyrotropin-releasing hormone, dopamine, and somatostatin (also known as growth hormone inhibiting hormone) [121]. Conversely, endocrine functions of magnocellular neurons in the ventrolateral part of the PVN project to the posterior pituitary, controlling the release of vasopressin and oxytocin [121]. In summary, the PVN is important in tying together aspects such as stress responses and energy homeostasis. Similar to the PVN, the AHA is also an important relay center controlling stress responses and energy homeostasis, housing both endocrine and autonomic responses [124]. The periventricular hypothalamic nucleus houses many kisspeptin neurons, responsible for the surge in LH and, in effect, GnRH release [125]. The anterior to central portions of the AHA are closest to and extend from the preoptic area, which regulates feeding and controls thermoregulation [126,127]. Thus, the AHA could be significant in combining stress and energy regulation. The periventricular hypothalamic nucleus is known to release, somatostatin and growth hormone, exhibiting sex differences in LH secretion [128], possibly due to differential ERβ expression between the sexes [129]. Thus, the periventricular hypothalamic nucleus is a major contributor to reproduction and gonadal functions. Furthermore, crosstalk occurs between the HPG and HPA axes, for example, sex differences are witnessed in the stress response and represent how gonadal steroid hormones modulate the HPA axis [30].

The mediobasal hypothalamus consisting of two main nuclei, the ARC and the VMH, is known as the metabolic control center responding to metabolic and psychologic stressors. ERα is extensively expressed in both the ARC and VMH to regulate energy homeostasis [120]. For example, females are able to lower their food intake in response to HFD through ERα-mediated actions in POMC neurons of the ARC [130]. It has been reported that the activity of these POMC neurons is increased by chronic restraint stress, which is associated with reduced activity of dopamine neurons in the ventral tegmental area [131]. Thus, the ARC is significant in regulating feeding and energy metabolism in respond to stress. In the VMH, ERα stimulates physical activity, basal metabolism, and brown adipose tissue thermogenesis, with no change in food intake [115]. Female mice that lack ERα in VMH neurons exhibit hypometabolism and abdominal obesity, but not hyperphagia [130]. Recently, it has been discovered that ERα expressed in these glucose sensing VMH neurons plays an important role in regulating blood glucose levels [132]. The VMH is significant in the neurocircuitry combining stress and energy regulation due to its roles in glucose-sensing and controlling glucose balance [133].

In summary, abovementioned are the major brain regions and associated neuropeptides involved in hypothalamic homeostatic regulation of metabolism in response to stress, with ERα-mediated mechanisms playing a central role.

#### 4.2.3. Organizational and Activational Effects of Estrogens

Sex chromosomes’ effects work alongside sex steroid hormones’ organizational and activational effects in the brain [134]. Besides sex hormones and sex chromosomes, sex differences in physiology, metabolism and behavior are also contributed by environmental and epigenetic factors.

During critical developmental periods, sex hormones exert important and permanent organizational effects on the development of brain structures that control sexually dimorphic neuroendocrine responses and behaviors [134]. Brain structure and circuitry are masculinized during brain development by androgens and estrogens at 10–20 weeks of pregnancy in humans, or from end of the embryonic period to the first postnatal day in rodents [135]. During the developmental organizational effects, components of male brain neural structures, pathways and circuitry are masculinized by the surge in testicular androgens that are aromatized to estrogens [136] via aromatase, an enzyme catalyzing the final step of estrogen synthesis [137]. Developing female rodent brains are protected from masculinization by α-fetoprotein that binds to maternal estrogens and forms a complex that does not cross the placenta [137]. Although less clear, it is thought that human female developing brains may be protected from masculinization by sex hormone-binding globulin [138]. In contrast to the permanent organizational effects of gonadal hormones on sex differentiation that occur during early developmental period, reservable activational effects occur during reproductive life stages. These activational effects modulate brain anatomic structures and circuitry developed during sexual differentiation to regulate neural activity between the sexes [134].

Thus, in addition to chromosomal effects, some sex divergence in physiology and behavior can be caused by organizational and/or activational effects of sex hormones. However, many underlying molecular mechanisms controlling sex differences in central regulation of metabolism and brain sexual differentiation remain unknown. For example, molecular mechanisms underlying brain cell differentiation, biogenesis, migration, and apoptosis promoted by sex hormones through direct and indirect pathways await elucidation and discovery [139]. Recent literature continues to uncover ERα-mediated mechanisms controlling sexually dimorphic energy homeostatic responses. As an example of one of the newest discoveries in the field, ERα in the VMH has been discovered recently to be responsible for repression of *reprimo* during male development and subsequent masculinization of this temperature regulation pathway in the VMH [140].

## 5. Conclusions

The search for knowledge in estrogen receptor neuroregulation of energy homeostasis is not a new endeavor, as the first discoveries on how ERα expression in the VMH contributes to obesity were made in 2007 [141]. Since then, the promise of nuclear and membrane ERs being a novel therapeutic drug target, such as development of selective estrogen receptor modulators (SERMs) and tissue selective estrogen complex (TSEC) combining SERM with one or more isoforms of estrogens [142], has emerged in benchwork literature and is growing greater over the years. Surprisingly, no ERα-targeted drug for obesity exists yet. However, a great deal of clinical research has focused on the roles of ER in promoting breast cancer or osteoporosis, and several FDA-approved drugs exist to this day such as tamoxifen, toremifene, raloxifene, and fulvestrant that target SERMs or selective downregulation of ER in mammillary cells [143,144]. The downside of these drugs is that they may produce breast cancer resistance effects, and therefore more work remains to increase their efficacy [145]. To summarize, although many neuroscience benchwork studies have uncovered the fine molecular actions of ER in the brain to increase energy expenditure and decrease food intake (e.g., [146]), it is curious that no FDA-approved SERM exists for treating metabolic disorders caused by dietary stress or physical stress. Perhaps our current understanding of ER mechanisms contains too many intricacies for easy development. For example, ER expression in some key brain nuclei controlling energy homeostasis is known to be changed by certain factors such as sex and diet [147]. On the other hand, a successful drug Wegovy was FDA-approved in 2021 for treating obesity [148], which is a receptor agonist for glucagon-like peptide 1, another commonly studied hormone for regulation of energy homeostasis.

Abundant literature has demonstrated that differential expression of sex hormone receptors, such as androgen receptor and ER, may lead to sex-distinct behavior and biological responses (e.g., [62]). Although this review focuses on the effects of estrogens through estrogen receptors, both estrogens and androgens contribute to sex differences in physiology and behavior. Furthermore, it is noteworthy that different underlying mechanisms between the sexes may still be at play even if phenotypic physiological and behavioral sex differences are not witnessed. For example, the dual-function hypothesis from De Varies [149] proposes that, although different brain structures between sexes have developed, levels of sex hormones or gene expression can be modified to compensate functional and behavioral differences [149]. Therefore, even if an overall sex difference in behavioral or functional phenotype is not evident, underlying mechanisms involving diverse signaling pathways may still differ, with opposing actions and thus abrogate sex differences, leading to similarity or even equivalence of overt phenotypes between the two sexes [149]. Mechanisms remain to be uncovered for the sex differences witnessed in physiological and behavioral effects due to metabolic and psychological stressors. Elucidation of these mechanisms will aid in development of important clinical indicators for better serving men and women who are suffering from metabolic or psychological stressors.

## Data Availability

Not applicable.

## Abbreviations

17α-E2: 17α-estradiol; 17β-E2: 17β-estradiol; ACTH: adrenocorticotropic hormone; AgRP: agouti-related protein; AHA: anterior hypothalamic area; α-MSH: α-melanocyte-stimulating hormone; ARC: arcuate nucleus of the hypothalamus; BLA: basolateral amygdala; BNST: bed nucleus of the stria terminalis; CEA: central nucleus of the amygdala; CNS: central nervous system; CRH: corticotropin releasing hormone; E1: estrone; E2: estradiol; E3: estriol; E4: estetrol; ER: estrogen receptor; ERE: estrogen responsive elements; FSH: follicular stimulating hormone; GABA: gamma-Aminobutyric acid; GnRH: gonadotropin releasing hormone; HFD: high-fat diet; HPA: hypothalamic–pituitary–adrenal; HPG: hypothalamic-pituitary-gonadal; KO: knockout; LH: luteinizing hormone; MC4R: melanocortin-4 receptor; mER: membrane estrogen receptor; NPY: neuropeptide Y; OFC: orbitofrontal cortex; PBN: parabrachial nucleus; PGC-1α: peroxisome proliferator-activated receptor gamma coactivator 1 alpha; POMC: pro-opiomelanocortin; PVN: paraventricular nucleus of the hypothalamus; RBA: relative binding affinity; SERM: selective estrogen receptor modulator; Sim1: single-minded 1; VMH: ventral medial nucleus of the hypothalamus.
